# Dauer Development Modulates Olfactory Behavior

**DOI:** 10.17912/micropub.biology.000479

**Published:** 2021-10-14

**Authors:** Johnny Vertiz, Heather Carstensen, Ray Hong

**Affiliations:** 1 Department of Biology, California State University, Northridge, Northridge, USA

## Abstract

Reproductive adults and developmentally arrested larvae often occupy different ecological niches and thus are expected to respond differently to environmental stimuli. To understand the genes that coordinate dauer development and olfactory behavior, we examined adult and dauer *C. elegans* in wild-type and dauer constitutive mutants (Daf-c). We found all dauers showed decreased attraction to all three odorants tested compared to adults, with *daf-7* dauer larva (DL) exhibiting a concentration-dependent preference shift towards isoamyl alcohol, suggesting that the TGF-ß pathway is involved in both dauer regulation and dauer-specific odortaxis.

**Figure 1. Olfactory responses of C. elegans dauer larvae:**
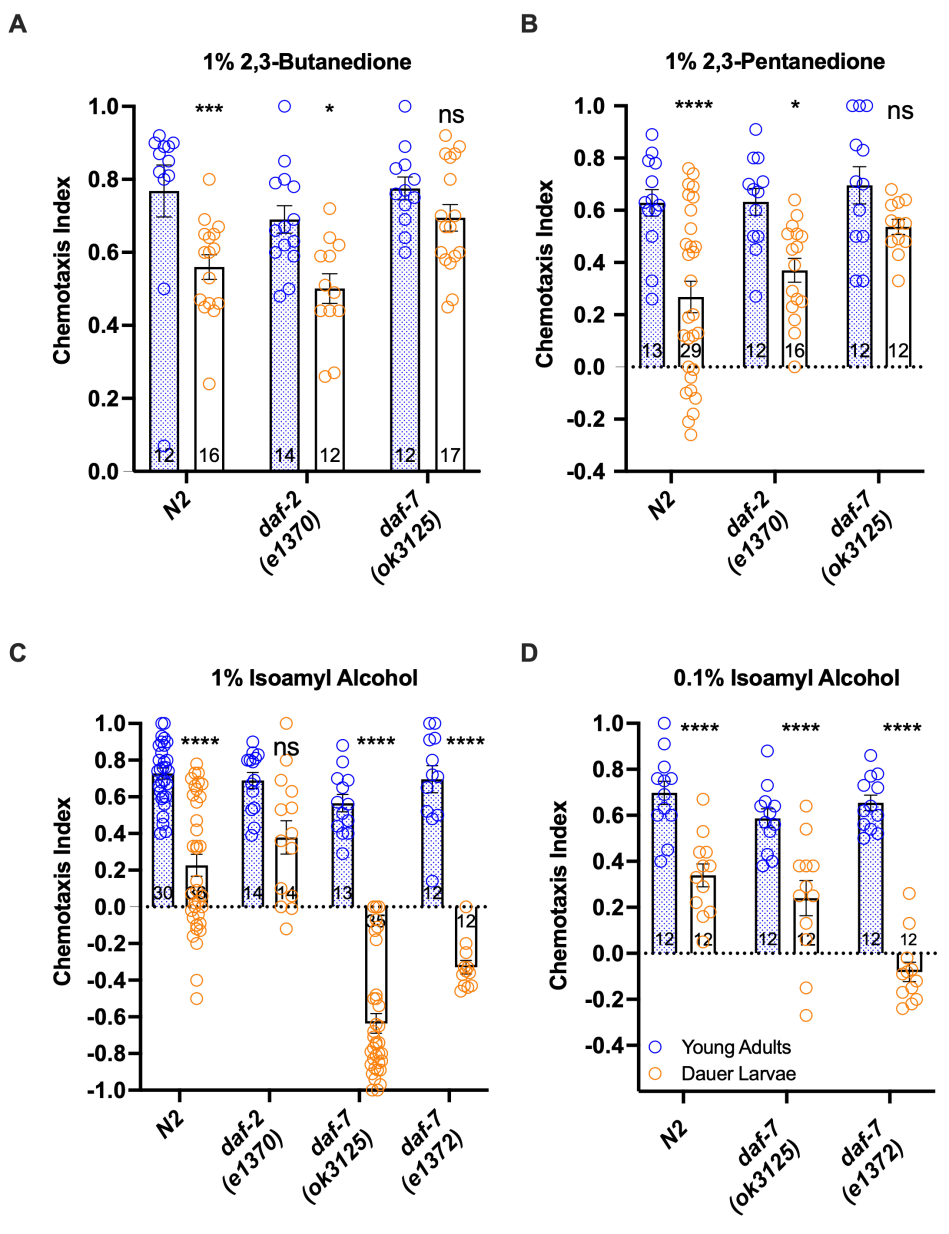
Chemotaxis between adults and dauer larvae differ significantly in response to **(A)** 1% 2,3-butanedione, **(B)** 1% 2,3-pentanedione, and **(C-D)** 1% and 0.1% isoamyl alcohol in wild-type N2, as well as in the Daf-c mutants *daf-2* and *daf-7.* For (A) and (C): One-way ANOVA between adults and dauer with Kruskal-Wallis test. Blue circles denote young adults and orange circles denote dauer larvae. For (B) and (D): Two-way ANOVA between adults and dauer with Sidak’s multiple comparisons test. *P<0.05, **P<0.01, ****P<0.0001, ns: not significant. Error bars indicate the standard error of the mean. The number of assays performed is indicated at the base of each bar.

## Description

In *C. elegans*, the dauer larva (DL) is a non-feeding and stress-resistant stage that can respond to environmental cues differently from adults. For example, *C. elegans* adults avoid CO_2_ while DL are attracted to CO_2_ (Hallem and Sternberg 2008; Hallem *et al.* 2011). Similarly, adults of the nematode *Pristionchus pacificus* are mildly attracted to a beetle host pheromone while its dispersive DL are highly attracted to the pheromone (Carstensen *et al.* 2021).

To determine if genes that affect dauer development also modulate olfactory behavior in *C. elegans*, we compared the odor response profiles of young adults versus DL in *C. elegans* of two well-studied Daf-c mutants, *daf-2* and *daf-7* *(*Riddle *et al.* 1981; Swanson and Riddle 1981; Kimura *et al.* 1997*)* towards three odorants. In *C. elegans* adults, the AWA neurons detect 2,3-butanedione (diacetyl), while the AWC neurons sense isoamyl alcohol (IAA) and 2,3-pentanedione (Sengupta *et al.* 1996; Chalasani *et al.* 2007). We found that wild-type DL showed less odor attraction than corresponding adults for 2,3-butanedione, 2,3-pentanedione, and IAA, consistent with the results of a previous study that included 2,3-butanedione (Hallem *et al.* 2011)(**Fig. 1A-C**). However, this adult-DL difference was not observed in *daf-2* towards IAA or *daf-7* towards 2,3-butanedione and 2,3-pentanedione. Furthermore, the response to IAA changed from attractive to repulsive in the DL of two *daf-7* alleles *(e1372, ok3125)*. To evaluate if the avoidance response to IAA is due to hypersensitivity to IAA in the *daf-7* DL, we also tested their response to a 10-fold lower IAA concentration (0.1%). We found that the strong avoidance response was no longer observed, and instead the dauer IAA attraction became significantly reduced in both *daf-7* alleles compared to adults, resembling the wildtype DL (**Fig. 1D**). In *C. elegans*, odor concentration-dependent preference shift has been observed for IAA for adult worms and is dependent on *odr-3* function (Yoshida *et al.* 2012). Thus, *C. elegans* wild-type and Daf-c DL showed decreased attraction compared to adults to all three odorants tested, with the *daf-7* mutations producing an accentuated concentration-dependent response to IAA in the DL, suggesting that the TGF-ß pathway is involved in both dauer regulation and dauer-specific response to IAA.

While remodeling of the AWC dendritic ends that increase their surface area have been speculated to heighten odor sensitivity in DL (Albert and Riddle 1988), our results show that only *daf-7* alleles exhibited a potentially higher sensitivity to isoamyl alcohol, while wild-type and *daf-2* DL were actually less attracted than adults to several odors. Mutation of the *daf-2* gene also mostly eliminated acute CO_2_ avoidance in adult *C. elegans*, while the *P. pacificus* Daf-c mutant *Ppa-hsd-2* exhibited greatly enhanced adult attraction to a host odor (Hallem and Sternberg 2008; Carstensen *et al.* 2021). These results suggest that dauer development has multiple effects on olfactory behavior, such that both wild-type and Daf-c DL should be assessed when surveying species-specific responses.

## Methods

Nematodes were cultured on OP50-seeded NGM plates and assayed at ~22°C. The chemotaxis assays for *C. elegans* were set up in a quadrant with worms loaded onto the center of 6 cm plates containing MOPS/Tween agar, along with two opposing pairs of odors and controls (Nuttley *et al.* 2002; Margie *et al.* 2013; Carstensen *et al.* 2021). 0.5 µl of 1 M sodium azide was spotted onto each odor or counter-attractant. Since *C. elegans* DL are hydrophilic, we collected DL primarily from the condensations present on the underside of the lids of recently starved plates by washing the lids with sterile spring water (Arrowhead, CA). For assays with Daf-c mutants, each plate was scored for either DL or YA using visual confirmation. Chemotaxis assays lasted 1-1.5 hours.

**Table:** 

**Strain**	**Genotype**
CB1370	*daf-2(e1370)III*
CB1372	*daf-7(e1372)III*
RB2302	*daf-7(ok3125)III*
N2	wildtype

## Reagents

MOPS buffer (pH 7)

Tween 20 (Polyoxyethylene 20-Sorbitan Monolaurate) CAS 9005-64-5

2,3-butanedione, isoamyl alcohol, and 2,3-pentanedione (Sigma-Aldrich)
